# Supplementary material for: The interactome of intact mitochondria by cross-linking mass spectrometry provides evidence for coexisting respiratory supercomplexes
Source: Mol Cell Proteomics. 2017 Dec 8;17(2):216–32. doi: 10.1074/mcp.RA117.000470 (PMC5795388; doi:10.1074/mcp.RA117.000470)
Supplement: Supplemental Data [file supp_RA117.000470_133922_0_supp_23978_fzffwf.zip › spectra_annotation/mito_spectra_annotation/368-1-12-1-5-1.pdf]

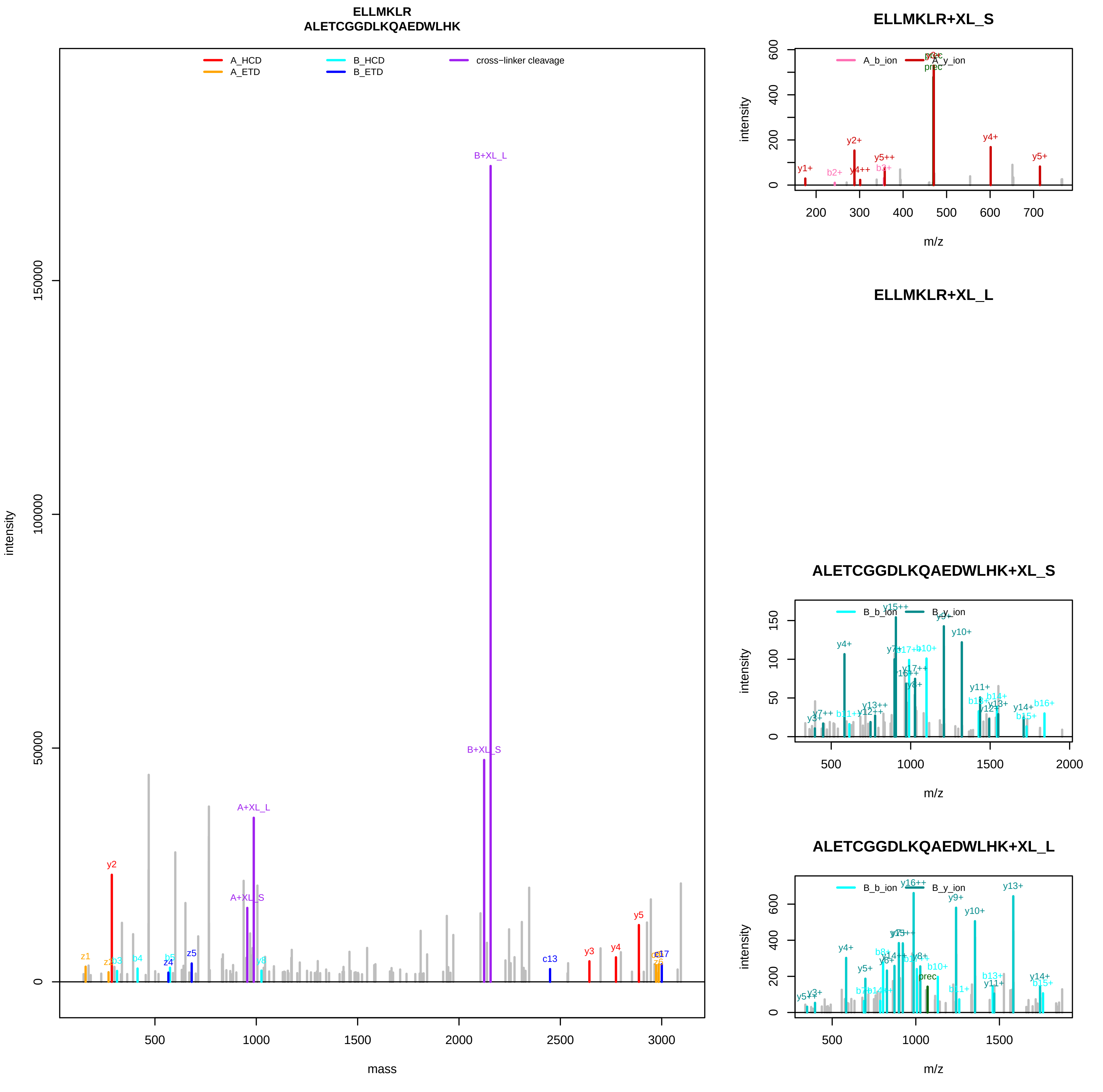
ELLMKLR
ALETCGGDLKQAEDWLHK
A_HCD
A_ETD
B_HCD
B_ETD
cross−linker cleavage
B+XL_L
B+XL_S
A+XL_L
A+XL_S
y2
y3
y4
y5
z1
z5
z4
b4
b5
c13
c17
intensity
mass
0
50000
100000
150000
500
1000
1500
2000
2500
3000
ELLMKLR+XL_S
A_b_ion
A_y_ion
prec
y1+
b2+
y2+
y4++
y5++
b3+
y4+
y5+
intensity
m/z
ELLMKLR+XL_L
ALETCGGDLKQAEDWLHK+XL_S
B_b_ion
B_y_ion
y15++
y9+
y10+
y4+
y11+
y14+
b16+
b15+
m/z
ALETCGGDLKQAEDWLHK+XL_L
B_b_ion
B_y_ion
y16++
y9+
y13+
y10+
y4+
y5+
prec
b10+
b11+
b13+
y11+
y14+
b15+
m/z
